# Downstream of tyrosine kinase/docking protein 6, as a novel substrate of tropomyosin-related kinase C receptor, is involved in neurotrophin 3-mediated neurite outgrowth in mouse cortex neurons

**DOI:** 10.1186/1741-7007-8-86

**Published:** 2010-06-18

**Authors:** Wei qi Li, Lei Shi, Yuan gang You, Yan hua Gong, Bin Yin, Jian gang Yuan, Xiao zhong Peng

**Affiliations:** 1 National Key Laboratory of Medical Molecular Biology, School of Basic Medicine, Peking Union Medical College, Institute of Basic Medical Sciences, Chinese Academy of Medical Sciences, Beijing, China; 2 Department of Cell Biology, Yale University, New Haven, CT, USA; 3 Department of Biochemistry, Medical College of Chinese People's Armed Police Force, Tianjin, China

## Abstract

**Background:**

The downstream of tyrosine kinase/docking protein (Dok) adaptor protein family has seven members, Dok1 to Dok7, that act as substrates of multiple receptor tyrosine kinase and non-receptor tyrosine kinase. The tropomyosin-related kinase (Trk) receptor family, which has three members (TrkA, TrkB and TrkC), are receptor tyrosine kinases that play pivotal roles in many stages of nervous system development, such as differentiation, migration, axon and dendrite projection and neuron patterning. Upon related neurotrophin growth factor stimulation, dimerisation and autophosphorylation of Trk receptors can occur, recruiting adaptor proteins to mediate signal transduction.

**Results:**

In this report, by using yeast two-hybrid assays, glutathione S-transferase (GST) precipitation assays and coimmunoprecipitation (Co-IP) experiments, we demonstrate that Dok6 selectively binds to the NPQY motif of TrkC through its phosphotyrosine-binding (PTB) domain in a kinase activity-dependent manner. We further confirmed their interaction by coimmunoprecipitation and colocalisation in E18.5 mouse cortex neurons, which provided more *in vivo *evidence. Next, we demonstrated that Dok6 is involved in neurite outgrowth in mouse cortex neurons via the RNAi method. Knockdown of Dok6 decreased neurite outgrowth in cortical neurons upon neurotrophin 3 (NT-3) stimulation.

**Conclusions:**

We conclude that Dok6 interacts with the NPQY motif of the TrkC receptor through its PTB domain in a kinase activity-dependent manner, and works as a novel substrate of the TrkC receptor involved in NT-3-mediated neurite outgrowth in mouse cortex neurons.

## Background

P62Dok/Dok1, which was identified by Carpino *et al*. and Yamanashi and Baltimore in 1997, was the first identified member of the downstream of tyrosine kinase/docking protein (Dok) family. Since then, six other members have been identified: p56Dok/Dok2/DokR, Dok3/DokL, Dok4, Dok5, Dok6/Dok5 like and Dok7 [1-4]. Dok family proteins are adaptor proteins that act as substrates of multiple receptor tyrosine kinases and non-receptor tyrosine kinases [5,6]. All seven family members share a similar structural topology: an N-terminal pleckstrin homology (PH) domain, a central phosphotyrosine-binding (PTB) domain, and a C-terminal region [4].

Interestingly, although all the members share almost same structural feature, they exert different, even opposite, roles in different tissues and organs. Dok1 to Dok3, which are predominantly expressed in haematopoietic tissues, act as negative regulators [7-12]. The results from single or double knockout of Dok1/Dok2 mice provided solid evidence to indicate their negative roles in leukemogenesis [11,13,14]. Yasuda *et al*. also showed that Dok1 and Dok2 are negative regulators of the T cell receptor signal pathway [15]. Dok3 exerts its negative role in the immunoreceptors of B cells and the macrophage signalling pathway by formation of Dok3-SHIP1 complex [1,16].

The other four family members, Dok4 to Dok7, are expressed in non-haematopoietic tissues, functioning in different places and in different ways. Dok4 is widely expressed, especially in intestine, kidney and lung that are all epithelial in origin. Bedirian *et al*. showed that Dok4 may function as an inhibitor of the tyrosine kinase signal pathway [17], while Grimm *et al*. found that Dok4 and Dok5 enhanced c-Ret-dependent activation of mitogen-activated protein kinase (MAPK) and promoted neurite outgrowth in PC12 cells [2]. Also, Uchida *et al*. showed that Dok4 may promote glial cell-derived neurotrophic factor (GDNF)-mediated neurite outgrowth in TGW cells through activation of the Rap1-Erk1/2 pathway [18].

Dok5 is mainly expressed in nervous system. It acts as a substrate for the insulin receptor (IR) and GDNF receptor c-Ret, and can promote GDNF-induced neurite outgrowth in PC12 cells [2,19]. For Dok6, *in situ *hybridisation results indicated that it is also specifically expressed in the nervous system, especially in the cortex and dorsal root ganglia (DRG). Crowder *et al*. showed that Dok6 could promote Ret-mediated neurite outgrowth in N2A-α1 cells [3]. Dok7 was identified recently and plays an important role in neuromuscular synaptogenesis by interaction with muscle specific kinase (MuSK). Mice lacking Dok7 fail to form acetylcholine receptor clusters and neuromuscular synapses [4]. Patients who have mutations in Dok7 develop congenital myasthenic syndrome (CMS) associated with small neuromuscular synapses [20,21].

Our previous work has shown that Dok5 is a substrate of tropomyosin-related kinase (Trk)B and TrkC receptors and is involved in neurotrophin-induced MAPK activation [22]. Neurotrophins, including nerve growth factor (NGF), brain-derived neurotrophic factor (BDNF), neurotrophin 3 (NT-3) and neurotrophin 4/5 (NT-4/5), and so on, are a family of growth factors implicated in several different functions in the nervous system [23,24]. There are two types of receptors for neurotrophins: the Trk family are high affinity receptors, while p75 is a low affinity receptor. The Trk family receptors have three members, TrkA, TrkB and TrkC, that are structurally conserved and exhibit specificity to different neurotrophins. TrkA is the preferred receptor for NGF, TrkB prefers BDNF, while NT-3 is the preferred ligand of TrkC [25]. Neurotrophins stimulation can induce the dimerisation and phosphorylation of Trk receptors, which then recruit and phosphorylate downstream adaptor proteins to mediate signal transduction [24-26]. It has been reported that neurotrophins and Trk receptors are involved in almost all stages of nervous system development, such as survival, proliferation, differentiation, migration, axon and dendrite projection, and neuron patterning [23,27-30].

To date, Dok6's functions in the nervous system have been poorly understood. In this study, we identified Dok6 as a novel substrate of the TrkC receptor. We confirmed that Dok6 selectively binds to the NPQY motif of TrkC through its PTB domain in a kinase activity-dependent manner. Immunostaining results showed that TrkC and Dok6 colocalise in E18.5 mouse cortical neurons. Knockdown of Dok6 decreased neurite outgrowth in cortical neurons upon NT-3 stimulation, which indicates that Dok6 is involved in NT-3-mediated neuronal development.

## Results

### Dok6 binds to the TrkC intracellular domain (ICD) but not TrkA or TrkB through the PTB domain in a yeast two-hybrid system

**Figure 1:**
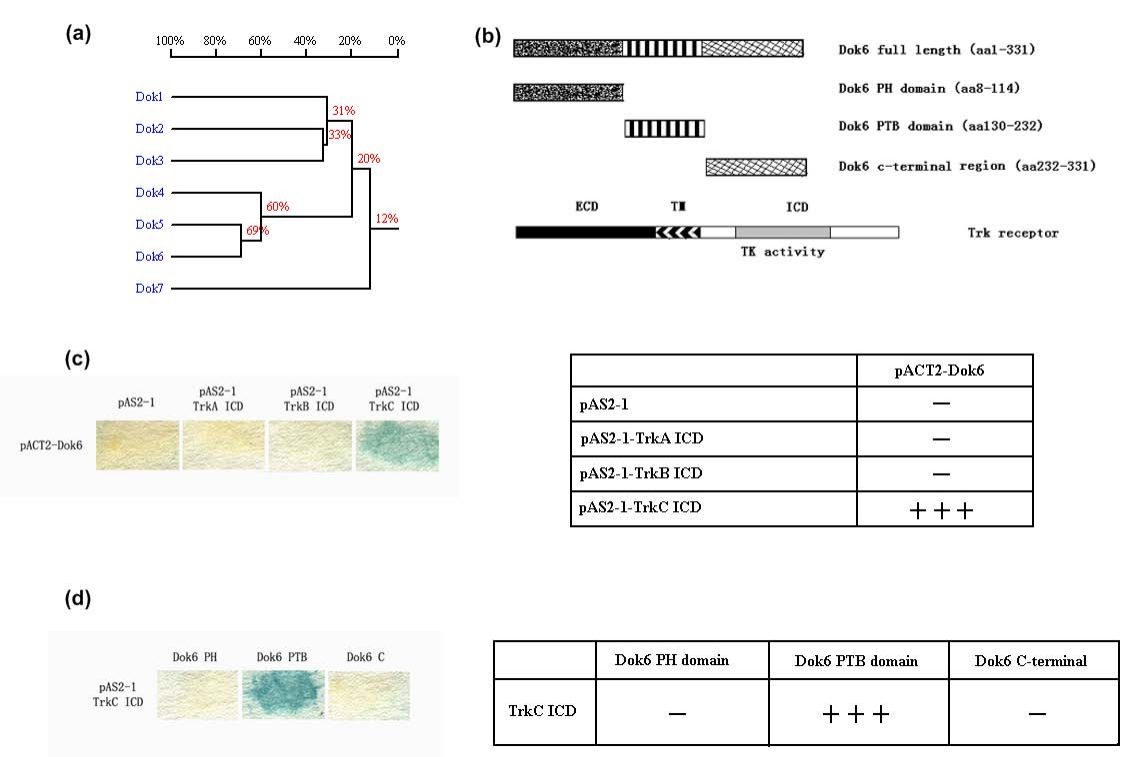
**Downstream of tyrosine kinase/docking protein (Dok)6 binds to tropomyosin-related kinase (Trk)C intracellular domain (ICD) but not TrkA or TrkB through the phosphotyrosine-binding (PTB) domain in a yeast two-hybrid system**. **(a) **Homology results of seven p62Dok family members analysed with DNAMAN software. **(b) **Schematic diagram showing the structures of the Dok6 and Trk receptors. **(c) **Dok6 interacts with the TrkC ICD, but not with TrkA ICD or TrkB ICD in a yeast two-hybrid system. Yeast (SFY526) were transformed with Trk ICD using a pAS2-1 vector and Dok6 using a pACT2 vector, and then cultured on SD/-Trp/-Leu plates for 4 days at 30°C. Colony lift β-galactosidase assays were performed according to the user manual. **(d) **The Dok6 PTB domain, but not the pleckstrin homology (PH) domain or C-terminal region, binds to TrkC ICD in yeast two-hybrid assays.

Our previous work indicated that Dok5, a member of the p62Dok family, directly interacted with TrkB and TrkC receptors to act as a downstream substrate to induce MAPK activation [22]. We used DNAMAN software (Lynnon Corporation, Pointe-Claire, Quebec, Canada H9R 2A3) to analyse the homology of Dok family proteins and found that Dok6 and Dok5 are the most conserved among all seven family members (Figure 1a), which means Dok6 may be more like Dok5 than any other members in the family. In consideration of the same specific neural expression pattern of Trk receptors and Dok6 in embryonic mice [3], we hypothesised that Dok6 may bind to Trk receptors directly to act as a downstream substrate, as Dok5 does, and play a role in the embryonic development. To verify this, we first used a yeast two-hybrid system to test whether there were interactions between Dok6 and Trk receptors. Interestingly, the results showed that Dok6 only binds to the TrkC ICD, but not to TrkA or TrkB, different to results with Dok5 (Figure 1b,c). The results implied more specificity of Dok6 to Trk receptors compared with Dok5. We then inserted the Dok6 PH domain, PTB domain and C-terminal region into the pACT2 vector and tested for these by yeast two-hybrid assay to determine the interaction region for Dok6. As shown in Figure 1d, like Dok5, the PTB domain of Dok6 is the key domain responsible for interaction; neither the PH domain nor the C-terminal region is involved in the interaction.

### Dok6 binds to TrkC receptor in glutathione S-transferase (GST) precipitation and coimmunoprecipitation (Co-IP) assays

**Figure 2:**
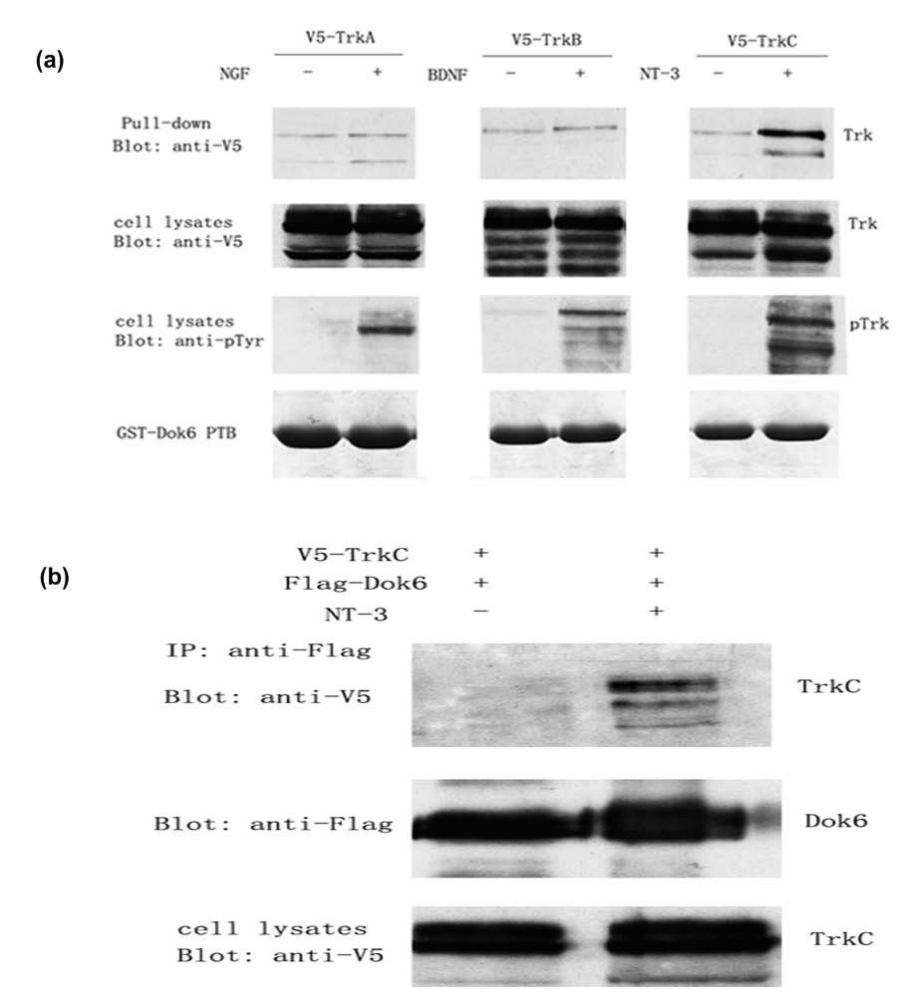
**Downstream of tyrosine kinase/docking protein (Dok)6 binds to tropomyosin-related kinase (Trk)C receptor in glutathione S-transferase (GST) precipitation and coimmunoprecipitation (Co-IP) assays**. **(a) **The Dok6 phosphotyrosine-binding (PTB) domain binds to the TrkC receptor in GST precipitation assays. Human embryonic kidney (HEK)293 cells were transfected with pcDNA3.1V5-Trks and treated with or without neurotrophins (100 ng/ml) for 10 min. The cell lysates were incubated with Dok6-PTB-GST Sepharose 4B beads at 4°C for 2 h. The bound proteins were then analysed by western blotting with anti-V5 antibody (upper panel). Cell lysates were examined by anti-V5 or anti-pTyr antibodies to detect the expression and phosphorylation levels of Trk receptors (second and third panel). Dok6-PTB-GST proteins were tested by Coomassie Brilliant Blue staining (bottom panel). **(b) **Dok6 binds to the TrkC receptor in Co-IP assays. HEK293 cells were cotransfected with Flag-Dok6 and V5-TrkC. Before harvest, the cells were treated with or without 100 ng/ml NT3 for 10 min. Cell lysates were immunoprecipitated by anti-Flag and then were detected by western blotting with anti-V5 and anti-Flag.

To confirm the results of the yeast two-hybrid assay, we performed a GST precipitation assay. As shown in Figure 2a, upon neurotrophin stimulation, Trk receptors became highly phosphorylated compared to those without stimulation, as shown by blotting with anti-p-Trk antibody (Figure 2a, panel 3). Consistent with results from the yeast two-hybrid assay findings, the precipitation results proved that the Dok6 PTB domain binds to the activated TrkC receptor strongly *in vitro*, while no remarkable binding was found between Dok6 and TrkA or TrkB with activation or no activation (Figure 2a, panel 1). Next, by Co-IP experiment, we further investigated the interaction between Dok6 and activated TrkC receptor (Figure 2b). The consistent results of the yeast two-hybrid assay, GST precipitation assay and Co-IP experiment implied that Dok6 selectively binds to TrkC receptor, but not TrkA or TrkB.

### Dok6 binds to the NPQY motif of TrkC receptor in a kinase activity-dependent manner and could be phosphorylated upon NT-3 stimulation

**Figure 3:**
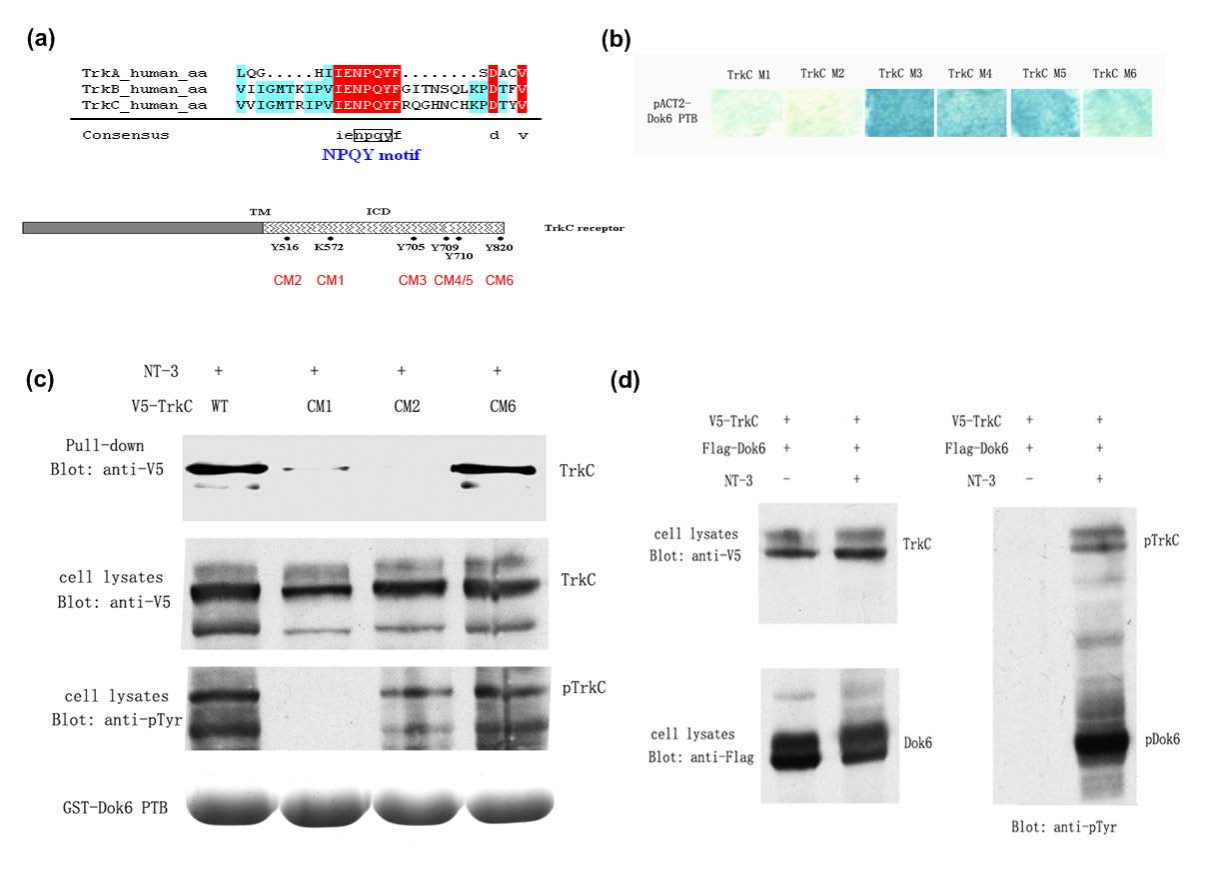
**Downstream of tyrosine kinase/docking protein (Dok)6 binds to the NPQY motif of tropomyosin-related kinase (Trk)C in a kinase-activity-dependent manner and was phosphorylated on neurotrophin 3 (NT-3) stimulation**. **(a) **Alignment of amino acid sequences around the NPQY motif of Trk family receptors (homology level: red, 100%; green, ≥50%), and schematic diagram showing the important amino acids of TrkC. **(b) **Mutation analysis of the key amino acids in the TrkC receptor for Dok6 binding. A yeast two-hybrid assay was performed with pACT2-Dok6-phosphotyrosine-binding (PTB) and TrkC intracellular domain (ICD) mutants using the pAS2-1 vector, including CM1(K572A), CM2(Y516F), CM3(Y705A), CM4(Y709D), CM5(Y710E), CM6(Y820F). **(c) **Dok6 binds to the NPQY motif of the TrkC receptor in a kinase activity-dependent manner in glutathione S-transferase (GST) precipitation assays. Human embryonic kidney (HEK)293 cells were transfected with wild-type TrkC, CM1, CM2 and CM6. After stimulation with NT-3, cell lysates were collected for GST precipitation assays. **(d) **Dok6 is phosphorylated by the TrkC receptor, which is activated upon NT-3 stimulation. HEK293 cells were cotransfected with V5-TrkC and Flag-Dok6, and cells were treated with/without NT-3 before harvest. Cell lysates were then analysed by western blotting with anti-pTyr antibody.

As shown in Figure 3a, there are five tyrosine residues (Tyr516 Tyr705 Tyr709 Tyr710 Tyr820) in the TrkC receptor ICD that could be phosphorylated upon treatment of NT-3; each tyrosine residue mutation would disrupt interaction between the receptor and corresponding substrates [25,31]. Among them, Tyr516 is located in the conserved NPQY motif, which is the typical binding motif of the PTB domain [26,31,32]. As known from previous work, the PTB domain recognises the NPQY motif in two different manners: dependent or independent on phosphorylation. Our previous work demonstrated that Dok5 binds to TrkB and TrkC directly at this site depending on the phosphorylation state. Therefore, we hypothesised that this tyrosine residue was also important for the interaction with Dok6. Another residue, Lys572, is essential for tyrosine kinase activity of TrkC. Mutation of this site would produce a kinase-inactive TrkC receptor that could be used to test whether this interaction is dependent on kinase activity or not. To this end, we constructed serial mutant TrkC receptors (including five tyrosine residue mutants and one lysine mutant) for yeast two-hybrid assay and GST precipitation experiments to determine the binding site on the TrkC receptor for Dok6 and to ascertain whether this binding depends on phosporylation or not.

As expected, the yeast two-hybrid results showed that TrkC Y516F (M2) lost most interaction with Dok6, while the other four tyrosine mutant receptors (M3-M6) did not dramatically affect binding. The kinase-inactive receptor TrkC K572A (M1) had no interaction with Dok6 either, which implied that the NPQY motif and the kinase activity of TrkC receptor are both essential for this interaction (Figure 3b).

Using GST precipitation experiments, we further confirmed that Dok6 binds to the NPQY motif of the TrkC receptor depending on phosphorylation. Wild-type TrkC receptor could be precipitated by Dok6 upon NT-3 stimulation, while neither the mutant receptor TrkC K572A (M1) nor TrkC Y516F (M2) could interact with Dok6 (Figure 3c). Consistent with yeast two-hybrid data, the unrelated tyrosine mutant TrkC Y820F (M6) showed similar result as the wild-type TrkC receptor. Moreover, as a substrate of TrkC, the phosphorylation level of Dok6 was greatly enhanced upon treatment with NT-3 (Figure 3d). In summary, the PTB domain of Dok6 binds to Tyr516 in the conserved NPQY motif of the TrkC receptor in a kinase activity-dependent manner.

### Dok6 colocalises with TrkC and binds to TrkC receptor in E18.5 mouse brains

**Figure 4:**
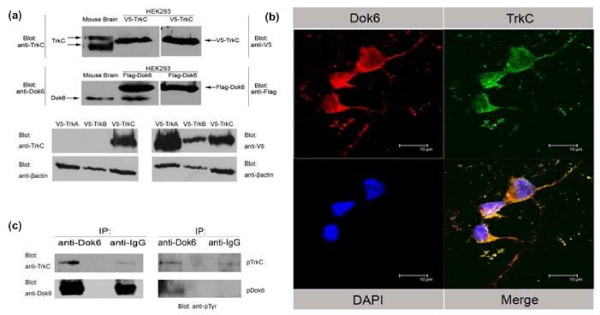
**Downstream of tyrosine kinase/docking protein (Dok)6 colocalises with tropomyosin-related kinase (Trk)C and binds to TrkC receptor in E18.5 mouse brains**. (a) Specificity tests for TrkC (R&D Systems) and Dok6 (Abcam) antibodies. Human embryonic kidney (HEK)293 cells were transfected with V5-TrkC and Flag-Dok6 separately. Cell lysates and homogenates of E18.5 mouse brains were then detected by western blotting with anti-TrkC and anti-Dok6 antibodies separately, using anti-V5 and anti-Flag antibodies as positive controls (panels 1 and 2). HEK293 cells were transfected with V5-TrkA, V5-TrkB and V5-TrkC separately. Cell lysates were then detected by western blotting with anti-TrkC and anti-V5 antibodies (panels 3 and 4). **(b) **TrkC colocalises with Dok6 in E18.5 mouse primary cortex cells. Immunostaining in cortex cells with anti-TrkC and anti-Dok6 antibodies. Cells were counterstained with 4',6-diamidino-2-phenylindole (DAPI) to show nuclei. Scale bar: 10 μm. **(c) **Dok6 binds to the TrkC receptor in mouse brain. Homogenates of E18.5 mouse brains were immunoprecipitated with anti-Dok6 and IgG antibodies; the bound proteins were then analysed by western blotting with anti-TrkC, anti-Dok6 and anti-pTyr antibodies.

As we had confirmed that Dok6 could interact with the TrkC receptor *in vitro*, next we investigated if Dok6 could bind to the TrkC receptor *in vivo*. To test TrkC and Dok6 commercial antibodies, we overexpressed V5-tagged TrkC and Flag-tagged Dok6 in human embryonic kidney (HEK)293 cells, and tested HEK293 cell lysates and E18.5 mouse brain lysates via western blot. As shown in Figure 4a, TrkC and Dok6 that was either endogenously expressed in E18.5 mouse brains or overexpressed in HEK293 cells (V5-TrkC, Flag-Dok6) were both recognised by these antibodies; the anti-V5 and anti-Flag antibodies were used as positive control (Figure 4a, panels 1 and 2). Additionally, to verify the specificity of the TrkC antibody, we overexpressed V5-tagged TrkA, TrkB and TrkC in HEK293 cells, and cell lysates were blotted with anti-V5 or anti-TrkC antibodies. The results showed that our anti-TrkC antibody only recognised the TrkC receptor, but not the TrkA or TrkB receptors (Figure 4a, panels 3 and 4).

As has been shown previously the expression levels of TrkC and Dok6 in embryonic mouse cortex are remarkable [2,3,28,30,33], so we hypothesised that Dok6 may work as a substrate of the TrkC receptor and play a role in embryonic mouse cortex development. Immunostaining showed that TrkC and Dok6 colocalise at the membrane, cytoplasm and neurites in primary cultured cortex neurons (Figure 4b). We then collected homogenates of E18.5 mouse brains to perform immunoprecipitation tests with anti-Dok6, using IgG as a negative control. The results showed that the TrkC receptor was immunoprecipitated by anti-Dok6 antibody remarkably well, but not by IgG (Figure 4c, left panel). Also, consistent with results achieved by *in vitro *experiments, both TrkC and Dok6 are phosphorylated (Figure 4c, right panel). Taken together, these results provided evidence that Dok6 works as a substrate of the TrkC receptor *in vivo *and may have important roles in cortical neurons.

### Dok6 plays a role in neurite outgrowth in E18.5 mouse primary cortex neurons

**Figure 5:**
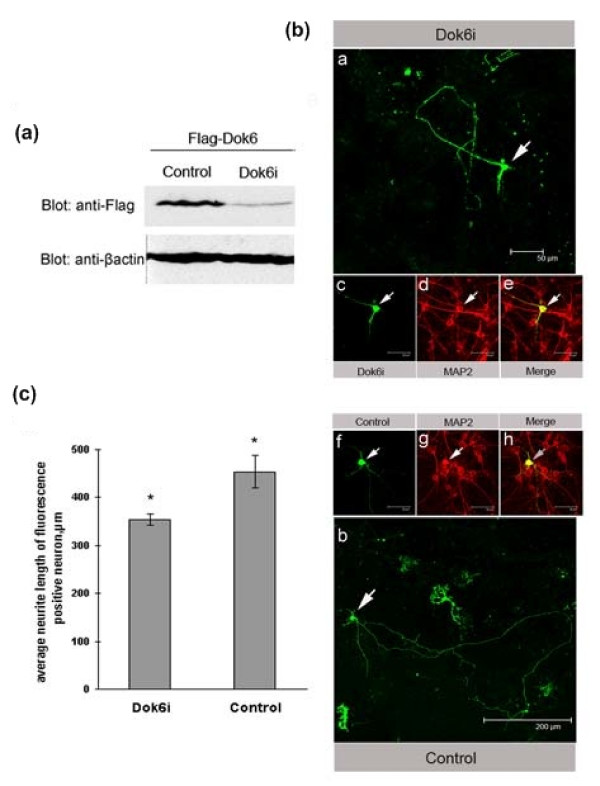
**Downstream of tyrosine kinase/docking protein (Dok)6 plays a role in neurite outgrowth in E18.5 mouse cortex primary neurons**. **(a) **Knockdown efficiency tests of Dok6i construct and nonsense control construct. Human embryonic kidney (HEK)293 cells were cotransfected with Flag-Dok6 and Dok6i or Flag-Dok6 and nonsense control constructs separately. The cell lysates were then analysed by western blotting with anti-Flag antibody. **(b) **E18.5 mouse cortex primary cells were transfected with Dok6i and nonsense control constructs, both of which were green fluorescence positive in cells. An immunofluorescence test was performed 48 h later with anti-microtubule-associated protein 2 (MAP2; red). and photographs of cells that were positive for both red and green fluorescence were taken (white arrow). Scale bars: panel a 50 μm, panel b 200 μm, panel c-h 30 μm. **(c) **Quantification of neurite outgrowth in cortex neurons. In all, 10 random fields of cells from each experimental group were photographed. Neurite length was quantified by measuring the longest neurite processes of cells that were positive for both green and red fluorescence by using NIS-Elements software. *P value < 0.05 by Student t test.

To study the exact roles of Dok6 in the cortex, we constructed knockdown clones of Dok6 (Dok6i) and nonsense controls (details are given in the Methods section). First, we tested the inhibition effect of our knockdown constructs by using western blotting. As shown in Figure 5a, Dok6i decreased the expression level of Dok6 almost threefold in HEK293 cells compared to the nonsense control construct.

Crewder *et al*. showed that Dok6 could promote neurite outgrowth in N2A cells [2,3], so we hypothesised whether Dok6 was also involved in neurite outgrowth in cortical neurons; this was tested for by knockdown of the expression of Dok6. We transfected primary cultured cortex cells (E18.5 ICR mouse) with Dok6i and nonsense control. As the RNAi vector had a green fluorescent protein (GFP) expression cassette within it, the cells with green fluorescence were therefore transfected cells; microtubule-associated protein 2 (MAP2; red) was used as a marker for neurons. Then, 48 h later, the lengths of neurites in cells with both red and green fluorescence (Figure 5b) were measured. The results showed that knockdown of Dok6 caused an almost 25% decrease in neurite outgrowth in primary cortex neurons compared to nonsense control (Figure 5c), which indicated that Dok6 plays a role in neurite outgrowth in cortical neurons.

### Dok6 is involved in cortex neuron neurite outgrowth through the NT-3/TrkC pathway

**Figure 6:**
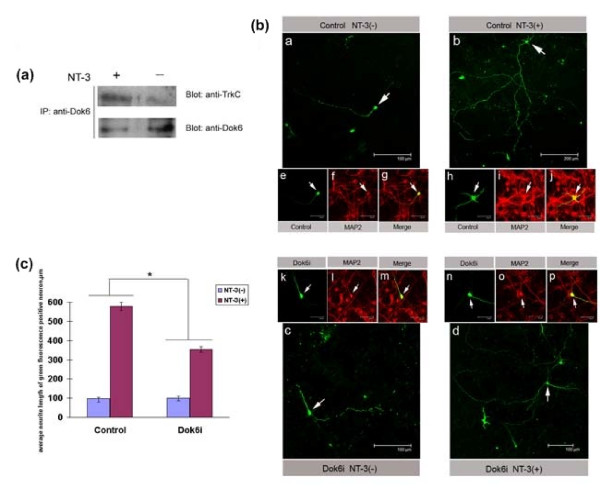
**Downstream of tyrosine kinase/docking protein (Dok)6 is involved in cortex neuron neurite outgrowth through the neurotrophin 3 (NT-3)/tropomyosin-related kinase (Trk)C pathway**. (a) Dok6 binds to the TrkC receptor in cortical neurons upon NT-3 stimulation. E18.5 mouse cortex neurons were cultured in empty DMEM for 24 h, then treated with or without 100 ng/ml NT-3 for 10 min. Cell lysates were immunoprecipitated by anti-Dok6 and were then detected by western blotting with anti-TrkC and anti-Dok6. **(b) **E18.5 mouse cortex primary cells were transfected with Dok6i and nonsense control constructs (green) separately; 12 h later, cultured cells were transferred into DMEM with/without 100 ng/ml NT-3 for another 72 h. Then immunofluorescence was performed with anti-microtubule-associated protein 2 (MAP2; red) and photographs of cells that were positive for both red and green fluorescence (white arrow) were taken. Panels a and b show the nonsense control group treated without or with 100 ng/ml NT-3; panels c and d show the Dok6i group treated without or with 100 ng/ml NT-3. Scale bars for panels a, c and d are 100 μm, panel b is 200 μm, and panels e-p are 30 μm. **(c) **Quantification of neurite outgrowth in cortex neurons. A total of 10 random fields of cells from each experimental group were photographed. Neurite length was quantified by measuring the longest neurite processes of cells that were positive for both green and red fluorescence by using NIS-Elements software. *P value < 0.05 by Student t test.

As we have shown that Dok6 works as a substrate of the TrkC receptor in mouse brain, next we investigated whether Dok6 exerted its role in cortex neuron neurite outgrowth through the NT-3/TrkC pathway. First, we performed Co-IP experiments to test for interactions between TrkC and Dok6 in cortex neurons that were treated with or without NT-3. Consistent with previous results, Dok6 binds to the TrkC receptor in cortex neurons upon NT-3 stimulation, while without NT-3 stimulation no obvious interactions exist (Figure 6a). Next, we transfected cortex cells with Dok6i and the nonsense control construct. Then, 12 h later, these cells were treated with or without 100 ng/ml NT-3. The neurite lengths of transfected cells were measured as described above (Figure 6b). The results showed that the average neurite length for positive neurons increased almost six fold after stimulation with NT-3 in the nonsense control group. However, in the Dok6 knockdown group, the increase was only about 3.5-fold (Figure 6c). This result suggests that NT-3 promotes neurite outgrowth in the cortical neuron, and knockdown of Dok6 partially blocks the NT-3 promotion effect in neurite outgrowth, which implies that Dok6 is exerting its role in cortex neuron neurite outgrowth through the NT-3/TrkC pathway.

## Discussion

In the present work we used yeast two-hybrid assays, GST precipitation assays and Co-IP experiments to demonstrate that Dok6 interacts with TrkC, which is a member of Trk receptors family. We also ascertained that Dok6 binds to the NPQY motif in the ICD of TrkC through its PTB domain, and this interaction depends on TrkC receptor kinase activity induced by NT-3. Our previous study showed that Dok5 could interact with TrkB and TrkC, but not with TrkA [22]. Although the homology between the Dok5 and Dok6 PTB domains is as high as 76.47%, there are still some different amino acids, which may result in the different protein structures. This may be why Dok5 and Dok6 exhibit different binding specificities to Trk receptor members. Additionally, the structural differences in Trk receptor members may be the reason why Dok6 only binds to TrkC, and not to TrkA or TrkB. Trk receptors play pivotal roles in almost all aspects of nervous system development, and knockout results for each member or its ligand indicate that TkrA, TrkB and TrkC play different roles in these events [24,25,27,28,34]. Dok6 selectively binding to the TrkC receptor may suggest its physiological function in the NT-3/TrkC pathway, while Dok5 may be involved in both the BDNF/TrkB and NT-3/TrkC pathways simultaneously.

It has been reported that many adaptor proteins may bind to the NPQY motif in Trk receptor ICDs through the PTB domain, such as Shc, insulin receptor substrate (IRS) and fibroblast growth factor receptor substrate 2 (FRS2) [35,36]. Trk receptors recruit these adaptor proteins to perform their pivotal roles. For example, upon stimulation with NGF, TrkA recruits Shc and phospholipase C (PLC)-γ1 to promote neurite outgrowth in PC12 cells [26]. Considering the complexity of the signal transduction network, it is hard to draw a picture of how these proteins could work together properly. In general, each single receptor could bind to several adaptor proteins; meanwhile, each adaptor protein could also interact with different kinds of receptor. Therefore, it is possible that, by using different receptor-adaptor combinations, cells can finely tune their signal transduction networks to fulfil different tasks. Also, we must consider the distinct expression patterns (time and space) of these receptors and adaptor proteins to study their exact roles *in vivo*. As reported by Crowder *et al*., Dok6 is mainly expressed in the embryonic brain and adult central nervous system, especially in the cortex and DRG [3]. This suggests that Dok6 may have a role in brain development. Additionally, the NT-3/TrkC pathway is believed to regulate neural precursor cell differentiation during cortical development [30]. The overlapping expression patterns of TrkC and Dok6 in the cortex imply their functional relevance on a physiological level. Furthermore, in this study our immunoprecipitation experiment and colocalisation test results confirmed that Dok6 interacts with TrkC in mouse cortex; this provided more evidence of Dok6's real function *in vivo*.

We also demonstrated that Dok6 is involved in NT-3-mediated neurite outgrowth of mouse cortex neurons, which indicates that Dok6 plays important roles in cortical development. These results enriched the signal transduction networks of the NT-3/TrkC pathway in the cortex. However, the downstream targets and signal pathway of Dok6 are poorly understood [2,3,19], and identification of these downstream targets is necessary for further studies. Like Dok1 to Dok5, several tyrosine residues localise in the C-termini of Dok6, which may serve as binding sites for downstream targets and hence may be critical for downstream signal transduction. However, although Dok5 and Dok6 share high homology with Dok1-Dok4, they fail to interact with adaptors such as Ras GTPase activating protein (RasGAP), Nck, and Src homology 2 domain-containing inositol phosphatase (SHIP), and so on, and bioinformatics studies have not clarified why this is the case [3,19]. It may be that immunoprecipitation, coupled with mass spectrometry identification assays, would be a good choice to elucidate the downstream targets. However, genetic study of Dok6 knockout mice may provide the strongest evidence for its physiological function *in vivo*.

## Conclusions

In summary, we have identified Dok6 as a new substrate of the TrkC receptor and Dok6 selectively binds to the NPQY motif of TrkC through its PTB domain in a kinase activity-dependent manner. We have also shown that Dok6, as a substrate of TrkC, plays an important role in NT-3-mediated neurite outgrowth in mouse cortex neurons. These results give new clues as to Dok6's function *in vivo*, and enriched the NT-3/TrkC pathway networks in cortical development. It also shed light on the treatment of nervous system deficiency diseases.

## Methods

### Antibodies and reagents

Antibodies used were: mouse anti-V5 monoclonal antibody (mAb) (Invitrogen USA), mouse anti-Flag mAb (Sigma USA), mouse anti-pTyr (PY99) mAb (Santa Cruz USA), goat anti-TrkC pAb (R&D Systems USA), rabbit anti-Dok6 polyclonal antibody (pAb) (Abcam England), mouse anti-MAP2 mAb (Abcam England). Human recombinant NT-3 was product of Peprotech Inc. Polylysine was purchased from Sigma.

### Plasmid construction

The human *trkA*, *trkB*, *trkC *and *dok6 *full-length cDNA were amplified from human foetal brain cDNA library. For the yeast two-hybrid assay, the *trk *receptor ICDs were cloned into the pAS2-1 vector, while the whole *dok6 *cDNA or sequences coding for the PH domain, PTB domain and C-terminal region were cloned into pACT2 vectors. The entire mutant constructs for the *trkC *receptor, including *trkC *K572A (M1), *trkC *Y516F (M2), *trkC *Y705A (M3), *trkC *Y709D (M4), *trkC *Y710E (M5) and *trkC *Y820F (M6) were generated by using a QuickChange Site-Directed Mutagenesis Kit (Stratagene).

For prokaryotic expression, the sequence coding for the *dok6 *PTB domain was cloned into the pGEX6P-1 vector. For eukaryotic expression, wild-type and mutant *trkC *receptors were cloned into the pcDNA3.1-V5 vector, while Dok6 was cloned into the pcDNA3.1-Flag vector.

For knockdown of Dok6, the Dok6i sequence (5'-GCACGAAAGATTAATGCTAGAT-3') and nonsense control sequence (5'-TTCTCCGAACGTGTCACGT-3') were constructed into the small hairpin (shRNA) vector pGPU6/GFP/Neo, which was purchased from Shanghai GenePharma.

### Cell culture and transfection

HEK 293 cells were maintained in Dulbecco's modified Eagle medium (DMEM) with 10% foetal bovine serum (Hyclone) and transfected using Lipofectamine 2000 (Invitrogen). E18.5 mouse cortex primary cells were cultured in Neurobasal-A medium (Gibco) with B27 (Gibco). All cells were maintained in a humidified atmosphere containing 5% CO_2_. For immunofluorescence, primary cortex cells were plated onto glass cover slips coated with 100 μg/ml polylysine, then transfected using Lipofectamine 2000 (Invitrogen).

### Yeast two-hybrid assay

*Trk *receptor ICDs were fused into the GAL4 DNA binding domain in the pAS2-1 vector. The wild-type *dok6 *or *dok6 *PH domain, PTB domain and C-terminal region were fused into the GAL4 activation domain in the pACT2 vector. The cotransformation was performed in yeast strain SFY526 by using the TE/LiAc (1 × TE/LiAC made fresh from stocks of 10 × TE [0.1 M Tris-HCI, 0.01 M EDTA, pH 7.5] and 10× LiAC [1 M lithium acetate adjusted to pH 7.5 with dilute acetic acid]) method according to the user manual for the MATCHMAKER yeast two-hybrid system 2 (Clontech USA). Transformed yeasts were selected on SD dropout medium/-Leu/-Trp plates at 30°C for 4 days. Then, colony lift β-galactosidase assays were performed.

### GST precipitation assay

The BL21 *Escherichia coli *strain was transformed with the pGEX6P-Dok6-PTB plasmid, then the overnight culture diluted 1:500 into 200 ml LB medium and shaken at 37°C until an OD_600 _value of 0.6 was reached. The induction was performed by shaking at 30°C for 4 h with 0.8 mM isopropyl-β-D-thiogalactopyranoside. The purified GST-Dok6-PTB fusion proteins were immobilised on Sepharose 4B beads according to the manufacturer's protocol (Amersham Biosciences). The HEK293 cells, which were transfected with serial pcDNA3.1-V5-Trk plasmids for 24 h, were treated with or without neurotrophin for 10 min. Cell lysates were centrifuged and then the supernatants were incubated with same amount of GST-Dok6-PTB Sepharose 4B beads at 4°C for 2 h. The beads were washed with 1 × phosphate-buffered saline (PBS) three times and then resuspended with 1 × SDS loading buffer. The denatured protein samples were analysed by western blotting and Coomassie Brilliant Blue staining.

### Co-IP assay

HEK293 cells were transfected into a six-well plate 1 day prior to assay and cultured to 90% density. Then, cotransfection was performed with pcDNA3.1-V5-TrkC and pcDNA3.1-Flag-Dok6 and 24 h later the cells were stimulated or not with 100 ng/ml NT-3 for 10 min. Then, cells were lysed with 750 μl lysis buffer and immunoprecipitation was performed as described previously [22].

### Immunoprecipitation

Animal care and experimental procedures conformed to Experimental Animal Regulations (China Science and Technology Commission Order no. 2) and were approved by the Institutional Animal Care and Use Committee at the Institute of Basic Medical Sciences, Chinese Academy of Medical Sciences and Peking Union Medical College. Homogenates of E18.5 ICR mouse brains were incubated with rabbit IgG (Zhongshan Goldenbridge Biotechnology Co., Ltd, Beijing, China) for 3 h at 4°C and then with protein A-agarose (Roche) overnight at 4°C. The next day, these homogenates were centrifuged at 1,000 *g *and the supernatants incubated with anti-Dok6 or IgG for 4 h at 4°C, followed by incubation with protein A-agarose for another 4 h at 4°C. Then, the samples were washed with PBS three times and resuspended with 1 × SDS loading buffer. The denatured protein samples were then analysed by western blotting with anti-TrkC and anti-Dok6.

### Immunofluorescence microscopy

E18.5 ICR mouse cortex primary cells were isolated and cultured as reported previously [37]. The cells were fixed in 4% paraformaldehyde for 15 min, followed by washes with PBS, and these cells were then permeabilised and blocked with PBS containing 0.1% Triton and 5% rabbit serum for 1 h. The cells were then incubated with primary antibodies diluted in blocking buffer overnight at 4°C, followed by incubation with secondary antibody for 2 h. After staining with 4',6-diamidino-2-phenylindole (DAPI), cover slips were mounted with mounting medium (Zhongshan Goldenbridge Biotechnology) and analysed using confocal microscopy.

### Neurite outgrowth assay

First, immunofluorescence (anti-MAP2, red fluorescence) was performed in E18.5 ICR mouse cortex primary cells transfected with knockdown constructs. Then, to measure neurite outgrowth, 10 random fields of cells from each experimental group were photographed. Neurite length was quantified by measuring the longest neurite process of cells that were positive with both green and red fluorescence by using NIS-Elements software (Nikon, 1300 Walt Whitman Road, Melville, N.Y. 11747-3064, USA)

## Authors' contributions

WL conceived and designed the study, carried out most of the collection, analysis and interpretation of data, and drafted the manuscript. LS contributed to the design of the study and the technical direction of the experiments, carried out part of the collection, analysis and interpretation of data. YY contributed to the design of the study and the technical direction of the experiments. YG and BY contributed to the design of the study and the revising of the manuscript. JY and XP contributed to the conception and design of the study, and the preparation of the manuscript. All authors read and approved the final manuscript.
